# Stresses and Disability in Depression across Gender

**DOI:** 10.1155/2014/735307

**Published:** 2014-01-21

**Authors:** Sharmishtha S. Deshpande, Bhalchandra Kalmegh, Poonam N. Patil, Madhav R. Ghate, Sanjeev Sarmukaddam, Vasudeo P. Paralikar

**Affiliations:** ^1^Department of Psychiatry, Smt. Kashibai Navale Medical College and General Hospital, Narhe, Pune, Maharashtra 411041, India; ^2^Maharashtra Institute of Mental Health, Pune 411001, India; ^3^Psychiatry Unit, KEM Hospital, Pune 411011, India

## Abstract

Depression, though generally episodic, results in lasting disability, distress, and burden. Rising prevalence of depression and suicide in the context of epidemiological transition demands more attention to social dimensions like gender related stresses, dysfunction, and their role in outcome of depression. Cross-sectional and follow-up assessment of men and women with depression at a psychiatric tertiary centre was undertaken to compare their illness characteristics including suicidal ideation, stresses, and functioning on GAF, SOFAS, and GARF scales (*N* = 107). We reassessed the patients on HDRS-17 after 6 weeks of treatment. Paired *t*-test and chi-square test of significance were used to compare the two groups, both before and after treatment. Interpersonal and marital stresses were reported more commonly by women (*P* < 0.001) and financial stresses by men (*P* < 0.001) though relational functioning was equally impaired in both. Women had suffered stresses for significantly longer duration (*P* = 0.0038). Men had more impairment in social and occupational functioning compared to females (*P* = 0.0062). History of suicide attempts was significantly associated with more severe depression and lower levels of functioning in case of females with untreated depression. Significant cross-gender differences in stresses, their duration, and types of dysfunction mandate focusing on these aspects over and above the criterion-based diagnosis.

## 1. Introduction 

Major depressive disorder (MDD) is a common and treatable psychiatric disorder with high morbidity and mortality. Complex interplay between mind, body, society, and culture has been implicated in the incompletely understood pathophysiology of depression [1]. Antecedent stresses and resulting disabilities interact with each other to worsen the depression, which in itself becomes additional stress. Patients' reports of stresses and adjustment problems frequently do not get due importance in understanding and managing depression, which may explain partial or delayed treatment response and frequent relapses.

Very few differences in the course of depression have been detected across gender. Factors like time taken for recovery, time for first occurrence, and number and severity of recurrences did not differ [2]. Background social contexts and support systems are important for understanding the contributing determinants of disease and for planning of treatment [3].

Depression has been identified as significant cause of morbidity and mortality [4]. Ferreira et al. (2013) [5] showed how rural India may be topping the list for depression and disability following organic disorders like stroke. Bromet et al. (2011) [6] in their exhaustive and impressive survey of cross-national epidemiology of depression showed how social conditions distinguish prevalence, disability, and burden of depression across ranges of income. Seedat et al. (2009) [7] showed the temporal narrowing of gender role traditionality in case of MDD among cohorts across the globe, which explains changing trends observed in Indian clinical practice and popular newspapers. Epidemiological transition [8, 9], globalization, and rapid urbanization [10] have contributed to changing social and family structure and function. Nuclear and small family norms have been responsible for changes in role responsibilities. However, generally family is a source of immense social support as well as of stress in Indian culture. Stresses experienced by males and females differ in nature and perception. Stress is known to precipitate or exacerbate episodes of depression. Mitigating stress by effective stress management has proved to be effective in prevention of MDD [11]. The nature of these stresses needs scrupulous assessment if preventive strategies have to be devised.

## 2. Subjects and Methods

This is a hospital-based study conducted at a tertiary hospital of a medical college. This hospital caters free medical services for the community. Patients predominantly from lower socioeconomic strata from in and around city, as well as from out-station, benefit from this hospital. A significant minority also comes from higher middle income social circles of the hospital staff.

Outpatients in psychiatry department clinically diagnosed to have depression were screened for inclusion in this study. Data collection was completed in a period of twelve months from January 2011 onwards. The study was completed over a period of two years.

Prior permission of ethics committee of the institute was obtained and informed written consent taken from each patient. Consecutive patients with depression (current major depressive episode, with or without dysthymic disorder and/or major depressive disorder) without significant biomedical illness were informed about the study and those willing to participate were included. A total of 107 such patients, 66 females and 41 males, were interviewed by authors (Sharmishtha S. Deshpande, Poonam N. Patil, and Bhalchandra Kalmegh) in the stipulated period of time. Findings about clinical characteristics of depression, presence of atypical features and comorbid psychiatric disorders in major depression from this study have been reported elsewhere [12]. This paper focuses on the gender differences in these patients' stresses and in functioning in various spheres.

After initial screening, SCID-I (Structured Clinical Interview for DSM IV axis I disorders) was administered to confirm the diagnosis of MDD [13]. HDRS (17 item Hamilton Depression Rating Scale) [14] was administered to note the severity of depression.

The patients were asked if they were undergoing any kind of stress in their life that may or may not be responsible for their depressed mood. Stresses reported by these patients were recorded verbatim (with duration) as perceived by these patients, which were later classified as per SCID-I axis IV guidelines and analyzed.

Their suicidal ideation was recorded using Paykel's scale [15]. The patient was then asked about any attempt of self-harm along with its details in the past. Any accidental poisoning or injury was also probed to rule out possibility of suicide attempt.

The patients were asked to follow up at 3 and 6 weeks for review. Attrition was significant and the patients often returned after symptom exacerbation following drug dropout. The number of patients taking regular treatment for six weeks or more (*N* = 40) was included in the second part of study. HDRS (17 item Hamilton Depression Rating Scale) was administered and level of functioning was also assessed in these patients.

Level of functioning was assessed and recorded with the help of GAF (Global Assessment of Functioning) scale, SOFAS (Social and Occupational Functioning Assessment Scale), and GARF (Global Assessment of Relational Functioning) scale. These are the scales for assessment and rating of axis V in DSM IV [16]. A semistructured questionnaire to score on SOFAS and GARF has been prepared by the author (Sharmishtha S. Deshpande) and used in an earlier study [17]. Interrater reliability of these scores has been reported in literature [18] and was also confirmed by the investigators in this study.

Data obtained was entered in Microsoft Excel sheets and later imported in suitable format for analysis. Bio-Medical Data Processor (BMDP 2.0) was used for statistical analysis. Data were analyzed for gender-wise comparison of various illness characteristics, namely, severity, stresses, suicidal ideation and functioning using *t*-test and chi-square tests of significance.

## 3. Results

This study assessed 107 patients with depression who were newly diagnosed to have depression or old patients who were off treatment for at least a month and had developed recurrence of symptoms. In addition, forty patients who had received regular treatment with antidepressants for six weeks or more were also reviewed for improvement in depressive symptoms and functioning.

Initial sample included 66 females and 41 males representing all age groups though mean age for females was higher than for males (Table 1). More males were single than females, whereas illiteracy and rural background in females were much higher compared to males. Patients coming from urban, semiurban, and rural areas were well represented in the sample. Majority of patients were servants or lower class workers and 40.9% women were homemakers, though we see representation of all types of occupations.

In Table 2 we see the improvement in mean HDRS score from 21 to 11.5. Mean HDRS score was comparable among males and females before as well as after treatment (Table 2). Mean duration of ongoing stress reported was much higher in women though duration of illness revealed from the history was comparable. The numbers of men and women with past history of suicide attempt as well as extent of suicidal ideation as measured by Paykel's score were comparable.

However, mean duration of suffered stress was significantly higher in females. This is the duration of prominent stress as reported by the patient during clinical interview (*P* = 0.0038). However, the nature of stresses perceived was significantly different as mentioned in Table 3.

Women in this study had frequent problems with primary support group than men (Table 3). Relationship problems in the family, especially with husband, were commoner (*P* < 0.001). Conflicts with in-laws and children were also reported by many women. Males reported financial problems as the commonest stress. This was not seen in case of women (*P* < 0.001). Problems related to social environment and occupational problems, though not statistically significant, were more among men. Also, other stresses and no stresses were reported more by men. Other stresses reported were academic failure, death of close relative, failure in romantic relationship, social isolation, and so forth.

When we considered the married subgroup of the sample, 5 out of 22 men and 39 out of 53 women had reported problems with primary support group (*χ* ^2^ = 14.5518, *P* = 0.00013). Table 3 also reflects on high number of women (31 out of 53 married women) and few men (4 out of 22 married men) reporting problems with spouse as the source of stress (*χ* ^2^ = 8.5942, *P* = 0.00337). The difference in the number of married men (13/22) and women (13/53) reporting economic problems was also statistically significant (*χ* ^2^ = 6.7448, *P* = 0.0094).

After 6 weeks of treatment, 38.4% women and 42.8% men patients were in remission (HDRS-17 score < 7). Among women with persistent depression, 43.7% reported interpersonal problems with spouse as predominant stressor and additional 31.2% variety of problems with other family members. Among women in remission, 70% had reported stress due to problems with children or daughters-in-law. Other sources of stress reported were own physical illness or financial problems, proportionately much less in either group. Half of the males in remission had not reported any stress, whereas the other half had stress due to separation from various family members. Among men with persistent depressive features, no stress was reported by 25%, financial stress by 37.5%, and some familial stress like dispute with brother, infertility, and separation from parents by the remaining 37.5%.

We have assessed social, occupational, and relational functioning of these patients in detail. Functioning of an individual in the sphere of household relationships is very important but seldom assessed systematically. Global Assessment of Relational Functioning scale (GARF) addresses issues like problem solving, organization, and emotional climate in the family which reflect on the quality of relationships. The functioning as a family rated from daily routines, warmth, and conflicts is rated in the range from optimal functioning to dysfunctionality, which are to be rated by psychiatrist in 100–0 scale. The scale has been used for a study in Brazil on validity of GARF; cut off of 70 was found to have acceptable validity coefficients [18].

Relational functioning (GARF score) was *maximally* affected functioning in this disorder, equally in males and females. It remained so even after treatment (score < 70) especially in case of females. Social and occupational functioning was more impaired in men than women at the time of presentation to psychiatrist. We see that 28 (42.4%) of women in this sample were housewives; many were working as maid, farmer, labourer, and so forth. They* seem to have* continued to do their work despite distress caused by illness. Global and mainly social and occupational functioning is affected significantly to a greater extent in males. When we compared GAF and GARF scores before and after 6 weeks of treatment by paired *t*-test, improvement was significant (*P* < 0.001) in males as well as females, which reflects on comparable response to treatment in terms of improvement in functioning in these spheres across gender. However, improvement in social and occupational functioning score (SOFAS) before and after treatment was significant in females (*P* < 0.001) but not significant in males (*P* = 0.120).

Women but not men with past history of self-harm had worse HDRS scores as well as worse functioning in all the spheres. This indicates further suicide risk in these patients and explains more frequency of suicide attempts among females. The time interval between suicide attempt and interview was variable from 1 month up to 10 years. However, it was comparable in males and females. Some patients also had history of more than one attempt.

After treatment scores for females show significantly higher HDRS score with past suicide attempt (16.85 +/− 8.21) than those without past history of suicide attempt (8.94 +/− 6.01). But the functioning scores were comparable to those without history of suicide attempt in females as well as males.

## 4. Discussion

In routine psychiatry practice, we see depressed men and women with varied stresses and differential impact of illness on their functioning. There seems to be intimate and complex interaction of these factors with suicidality in this disorder. This study attempted to objectively assess this relationship.

In the last three decades suicide rate has increased by 43% in India and ratio of males to females is stable at 1.4 : 1 [19]. Major depression is the commonest among contributors. Importance of social and public health aspects of suicide prevention in India has been emphasized in literature [20, 21]. Gender differences in suicide attempters are succinctly reported in a study from South India [22]. Females were more illiterate, rural and had less psychopathology. Significant numbers of females in our study were also from rural background and illiterate, but they reported suffering from stresses for significantly longer time (Table 1). This sample had patients with varied educational level and occupations represented, which can be seen as a limitation of this study. Comparable illness characteristics in males and females have been confirmed (Table 2) as reported in previous studies [2, 23–25]. However, females readily affirmed stresses, especially in the family domain (Table 3). Duration of these stresses was significantly prolonged in females as seen in Table 2. This could be due to more sensitivity of women towards problems in relationships as well as a function of dual importance of home in their life—as a work place and as an intimate group.

Social factors in depression have been extensively studied. Social variables are reported to differ across gender though there is no difference in overall course or severity of depression [2, 23–25]. This has been confirmed in the present study.

Long term follow-up studies in women have reported persistent effect of depression on their social interactions and relationships [25]. We see modest degree of improvement in mean scores of social-occupational, relational, and global functioning after treatment of depression (Table 4). This study assessed functioning using DSM-IV axis V scales. SOFAS and GARF are different constructs but related to GAF [14]. GARF has been correlated with clinician-assessed axis II pathology [26]. These scales are useful in assessing changes in personality psychopathology, social adjustments, and relationship skills. All these are important in prognosis and outcome of major depressive disorder.

Gender differences in stress experiences and stress vulnerability have been elaborately discussed by Eisenberg [1], in which findings mainly indicate protective effect of marriage against depression in males, but more negative effect on females. In animal research the opposite effect of same stress on male and female hippocampus has been documented indicating probable biological aspect of these findings [27].

Recent large systematic study “Predict D” has reported that preventive measures will achieve a greater reduction in the prevalence of depression than measures undertaken to eliminate risk factors after onset [7]. Our study has highlighted perceived stresses of local Indian populations and their differential impact across genders and marital status.

In an Indian study, significantly more women with features of neurasthenia spectrum disorder had identified marital problem as the most important perceived cause for their suffering, while none of the men did so [28]. Similar results from this study can be explained as patients are from the similar cultural background (Table 3).

Though depression is the third leading cause of disability, it is seldom quantified. We measured it clinically (HDRS) and also indirectly in terms of functional impairment (Table 4). In a 10-year follow-up study of depression, impairment in social functioning (housework, social interactions, and leisure) was known to persist for years after recovery though work functioning improved significantly [29]. Functional disability in females, especially housewives, is seldom noticed till it reaches significant proportions. Though they suffer great distress, they continue to do household work and try their best to conform to role expectations embodied by social norms. Concern about conforming to social expectations in females is also reported in other studies [30]. Difficulties in social and relational functioning are likely to lead to secondary distress in women which could be the perpetuating factors for depression and suicidality.

In our study 24 (60%) patients had failed to achieve remission after treatment (HDRS scores > 7). Yet their functioning (axis V scores) had improved statistically significantly. This may explain the observation that risk of self-harm persists after apparent clinical improvement.

As the concept of quality of life often differs across various sociocultural groups, assessing their functioning in various spheres seems to be more pertinent. Also, it is hard to find indigenously validated quality of life scales for depression suitable across heterogeneous patient groups in diverse Indian culture. Most Indian women are also known to equate their quality of life with their perceived adequacy in rolefunctioning [19].

In an Indian study, the most common reasons for suicidal attempts were marital and interpersonal problems followed by psychiatric and physical illnesses in females [21]. 71% of suicide attempters in India are below the age of 44 years [21]. Assessment of relational functioning is thus crucial in MDD patients. We see that this was the most affected sphere of functioning and least to improve with routine outpatient treatment for depression (Table 4). Though very few males reported stresses in the primary support group (Table 3), their GARF score is comparable with females as depicted in this table. This could also be due to underreporting of relational stresses by men. The relational functioning is likely to have worsened due to depressed or irritable mood and resultant depressive cognitions. It needs to be explored further by undertaking a longitudinal study to see if enhancing relational functioning helps in improving depressive features in either gender.

Severity of depression and dysfunction in women with history of suicide attempts are higher as depicted in our study (Table 5) highlighting that establishing diagnosis on axis I is not equivalent to full description of psychopathology. Role of axis IV (stresses) and V (functioning) in perpetuating axis I disorder needs to be understood individually in every patient with depression.

**Figure 1:**
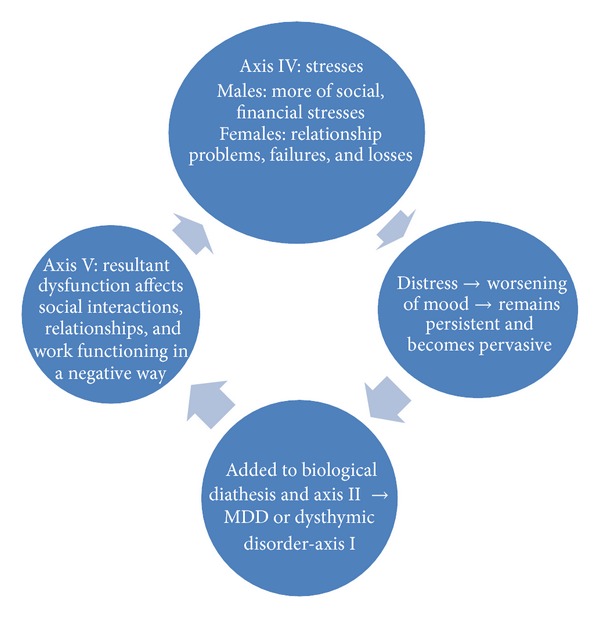
Interactive model of axes I, IV, and V in depression.

Limited success in managing depression with sole pharmacotherapy has been mentioned in literature [31] and so also more favourable outcome with combined use of psychotherapy with pharmacotherapy [32]. Review of contextual interventions for MDD has revealed significant success in 8 out of 13 studies reviewed recently [33]. The interpersonal problems especially in depressed patients living with a partner have been reported to be successfully managed by group couples' therapy, with lesser number of dropouts in the London Depression Intervention Trial [34]. Such management needs to be a part of regular treatment of depression in Indian setting. Considering interactions between axes I, IV, and V for appropriate psychosocial intervention along with drug treatment is necessary for comprehensive use of biopsychosocial model in diagnosis and management.  Figure 1 shows the interactive model of axes I, IV, and V in depression.

*Limitations of the Study.* Patients across wide age range, educational levels, different occupations, and marital status are included in the sample reducing its homogeneity.

Limited duration of followup (6 weeks) and small size of after treatment group of patients (*N* = 40) limit generalization of findings.

## 5. Conclusion 

There are qualitative and quantitative differences in stresses experienced by males and females that may have both cause and effect relationship with depression via resultant dysfunction. One should consider causative role of functioning in the complex etiopathology of depression in the context of body, mind, society, and culture. Gender differences in stresses and various spheres of functioning should be addressed in routine clinical practice. Relational functioning, being the most affected domain, should be assessed regularly in depressed patients.

Women have often suffered chronic stress commonly due to problems with primary support group. Mental health professionals should attend to these problems for necessary intervention. More proactive approach by clinicians to enhance social and relational functioning appears important in suicide prevention.

Distinct differences across gender in stresses and functioning in depression warrant longitudinal studies to explain the directionality between functioning, stress, and depression.

## Figures and Tables

**Table 1:** Sociodemographic characteristics of the 107 patients with MDD.

Demographic characteristic	Females *n* = 66 (%)	Males *n* = 41 (%)
Mean age (S.D.)	37.18 (11.69)	33.09 (10.09)
Residence		
Urban	27 (40.9)	26 (63.4)
Semiurban	23 (34.8)	8 (19.5)
Rural	16 (24.3)	7 (17.1)
Education		
Illiterate	19 (28.8)	1 (2.5)
Up to 10th	33 (50.0)	23 (56.1)
Up to college graduation	12 (18.2)	8 (19.5)
Graduate and above	2 (3.03)	9 (21.9)
Marital status		
Married	53 (80.2)	22 (53.6)
Divorced/separated/widowed	8 (12.2)	3 (7.3)
Unmarried	5 (7.6)	16 (39.1)
Occupational status		
Student	5 (7.6)	6 (14.6)
Maid/servant	16 (24.3)	6 (14.6)
Own business	15 (22.7)	17 (41.5)
Service/professional	3 (4.5)	8 (19.5)
Homemaker	27 (40.9)	0
Unemployed	0	4 (9.8)

**Table 2:** Comparison of duration of stress and illness characteristics across gender.

Illness characteristic	Females	Males	“*P*” by *t*-test and statistical significance
Mean duration of total illness (months) (S.D.) Females (*n* = 66), males (*n* = 41)	29.36 (34.41)	37.07 (50.15)	0.348 Not significant
Mean duration of current episode (months) (S.D.) Females (*n* = 66), males (*n* = 41)	12.83 (22.13)	10.73 (12.65)	0.581 Not significant
Mean duration of stress (months) (S.D.) Females (*n* = 66), males (*n* = 41)	*64.3 (85.35) *	*22.32 (40.12) *	*0.004* *Highly significant *
Mean of total number of episodes (S.D.) Females (*n* = 66), males (*n* = 41)	1.37 (0.74)	1.40 (0.78)	0.840 Not significant
Mean Paykel suicidal ideation score (S.D.) Females (*n* = 66), males (*n* = 41)	1.03 (0.91)	1.12 (0.90)	0.612 Not significant
Mean HDRS (S.D.) Females (*n* = 66), males (*n* = 41)	21.07 (5.26)	20.93 (6.36)	0.896 Not significant
Mean HDRS (S.D.) [after treatment] [*N* = 40] Females (*n* = 25), males (*n* = 15)	11.4 (7.18)	11.86 (9.80)	0.173 Not significant
Past history of suicide attempt (before treatment group) Females (*n* = 66), males (*n* = 41)	11 (16.7)	7 (17.0)	Chi-square = 0.0446 *P* = 0.8327 Not significant
Past history of suicide attempt (after treatment group) [*N* = 40] Females (*n* = 25), males (*n* = 15)	7 (28)	4 (26.6)	Chi-square = 0.0752 *P* = 0.7838 Not significant

**Table 3:** Various stresses reported by male and female patients with MDD categorized as per axis IV from SCID I.

Stress grouped as per axis IV categories in SCID I	Females *n* = 66 (%)	Males *n* = 41 (%)	Chi-square Test statistic, *P*, or Fisher's exact *P* and statistical significance
Problems with primary support group	*51 (77.3) *	*10 (24.4) *	*Chi-square = 28.868* *P* < 0.001 *Highly significant *
Spouse	*31 *	*4 *	
Children	*12 *	*2 *	
Parents/sibs	*3 *	*3 *	
In-laws/others	*5 *	*1 *	
Problems related to social environment	12 (18.2)	10 (24.4)	Chi-square = 0.597 *P* = 0.440 Not significant
Educational problems	1 (1.5)	0	Fisher's exact *P* = 0.999 Not significant
Occupational problems	1 (1.5)	3 (7.3)	Fisher's exact *P* = 0.156 Not significant
Housing problems	0	0	Fisher's exact *P* = 1.0 Not significant
Economic problems	*12 (18.2) *	*27 (65.8) *	*Chi-square = 24.812* *P* < 0.001 *Highly significant *
Problems with access to healthcare services	3 (4.5)	0	Fisher's exact *P* = 0.284 Not significant
Problems related to interaction with legal services or crime	2 (3)	1 (2.4)	Fisher's exact *P* = 1.0 Not significant
Other psychosocial problems	3 (4.5)	3 (7.3)	Fisher's exact *P* = 0.673 Not significant
No stresses reported	7 (10.6)	7 (17.1)	Chi-square = 0.930 *P* = 0.335 Not significant
Multiple stresses reported	23 (34.8)	14 (34.1)	Chi-square = 0.005 *P* = 0.941 Not significant
Past history of suicide attempt (before treatment group)	11 (16.7)	7 (17.0)	Chi-square = 0.0446 *P* = 0.8327 Not significant

**Table 4:** Scores of level of functioning in major depressive disorder across gender.

Scores on various functioning scales (axis V)	Before treatment			After treatment		
	Mean score (SD) Females (*n* = 66)	Mean score (SD) Males (*n* = 41)	*P* by *t*-test and stat. significance	Mean score (SD) Females (*n* = 25)	Mean score (SD) Males (*n* = 15)	*P* by *t*-test and stat. significance
GAF	59.01 (9.37)	55.75 (8.97)	*0.078* *Marginally significant *	69.2 (9.43)	69 (12.28)	0.954 Not significant
SOFAS	59.24 (8.51)	54.63 (7.94)	*0.006* *Highly significant *	68.6 (10.66)	64.33 (13.21)	0.269 Not significant
GARF	52.50 (13.54)	52.80 (11.01)	0.904 Not significant	65.8 (17.54)	67.7 (14.49)	0.726 Not significant

**Table 5:** Association between suicide attempt and scores of psychopathology and functioning.

Axis V score	Women with history of past suicide attempt (*n* = 11) Mean (S.D.)	Comparison with women without history of suicide attempt (*n* = 55) (“*t*,” *P*, and statistical significance)	Men with history of past suicide attempt (*n* = 7) Mean (S.D.)	Comparison with men without history of suicide attempt (*n* = 34) (“*t*,” *P*, and statistical significance)
GAF	50.0000 (13.7840)	*3.8474, 0.0002; highly significant *	53.00 (6.30)	0.8905, 0.38 Not significant
SOFAS	51.8181 (9.5584)	*3.4185, 0.0010; highly significant *	54.28 (6.72)	0.1259, 0.9 Not significant
GARF	42.7273 (11.9087)	*2.7522, 0.0076; highly significant *	53.57 (4.76)	−1.997, 0.84 Not significant
HDRS	25.7272 (4.9008)	*−3.4734, 0.0009; highly significant *	21.00 (5.13)	−0.0329, 0.97 Not significant

## References

[1] Eisenberg L (1977). Psychiatry and society. A sociobiologic synthesis. *The New England Journal of Medicine*.

[2] Simpson HB, Nee JC, Endicott J (1997). First-episode major depression: few sex differences in course. *Archives of General Psychiatry*.

[3] Engel GL (1977). The need for a new medical model: a challenge for biomedicine. *Science*.

[4] Collins PY, Patel V, Joesti SS, March D, Insel TR, Daar AS Grand challenges in global mental health: a consortium of researchers, advocates and clinicians announces here research priorities for improving the lives of people with mental illness around the world, and calls for urgent action and investment. *Nature*.

[5] Ferreira  L, Ferreira Santos-Galduróz  R, Ferri CP, Fernandes Galduróz JC (2013). Rate of cognitive decline in relation to sex after 60 years-of-age: a systematic review. *Geriatrics & Gerontology International*.

[6] Bromet E, Andrade LH, Hwang I (2011). Cross-national epidemiology of DSM-IV major depressive episode. *BMC Medicine*.

[7] Seedat S, Scott KM, Angermeyer MC (2009). Cross-national associations between gender and mental disorders in the World Health Organization World Mental Health Surveys. *Archives of General Psychiatry*.

[8] Manton KG (1988). The global impact of noncommunicable diseases: estimates and projections. *World Health Statistics Quarterly*.

[9] Maire B, Lioret S, Gartner A, Delpeuch F (2002). Nutritional transition and non-communicable diet-related chronic diseases in developing countries. *Sante*.

[10] Vlahov D, Galea S, Gibble E, Freudenberg N (2005). Perspectives on urban conditions and population health. *Cadernos de Saúde Pública*.

[11] Bottomley C, Nazareth I, Torres-González F (2010). Comparison of risk factors for the onset and maintenance of depression. *The British Journal of Psychiatry*.

[12] Deshpande SS, Patil P, Kalmegh B, Ghate MR (2013). Untreated major depressive disorder with and without atypical features: a clinical comparative study. *Asian Journal of Psychiatry*.

[13] First MB, Gibbon M, Spitze RL, Williams: JBW (2002). *User’s Guide for the Structured Clinical Interview for DSM-IV-TR Axis I Disorders-Research Version-(SCID-I for DSM IV TR, November 2002 Revision)*.

[14] Hamilton M (1960). A rating scale for depression. *Journal of Neurology, Neurosurgery, and Psychiatry*.

[15] Paykel ES, Myers JK, Lindenthal JJ, Tanner J (1974). Suicidal feelings in the general population: a prevalence study. *British Journal of Psychiatry*.

[16] American Psychiatric Association publication (2005). Mood disorders. *Quick Reference to the Diagnostic Criteria From DSM-IV TR, 2000*.

[17] Gaikwad R, Deshpande S, Raje S, Dhamdhere DV, Ghate MR (2006). Evaluation of functional impairment in psoriasis. *Indian Journal of Dermatology, Venereology and Leprology*.

[18] Hilsenroth MJ, Ackerman SJ, Blagys MD (2000). Reliability and validity of DSM-IV axis V. *The American Journal of Psychiatry*.

[19] Vijaykumar L (2010). Indian research on suicide. *Indian Journal of Psychiatry*.

[20] Venkoba Rao A, Mahendran N, Gopalakrishnan C (1989). One hundred female burn cases: a study in suicidology. *Indian Journal of Psychiatry*.

[21] Jena S, Siddhartha T (2004). Non-fatal suicidal behavior in adolescents. *Indian Journal of Psychiatry*.

[22] Sudhir Kumar CT, Mohan R, Ranjith G, Chandrasekaran R (2006). Gender differences in medically serious suicide attempts: a study from South India. *Psychiatry Research*.

[23] Zlotnick C, Shea MT, Pilkonis PA, Elkin I, Ryan C (1996). Gender, type of treatment, dysfunctional attitudes, social support, life events, and depressive symptoms over naturalistic follow-up. *American Journal of Psychiatry*.

[24] Wu P-C (2010). Measurement invariance and latent mean differences of the beck depression inventory ii across gender groups. *Journal of Psychoeducational Assessment*.

[25] Nolen-Hoeksema S (2001). Gender differences in depression. *Current Directions in Psychological Science*.

[26] Feijo Mello A, Blay LS, Kohn R (2007). Global assessment of relational functioning scale (GARF): a validity study in patients with recurrent major depression in Brazil. *Transcultural Psychiatry*.

[27] Shors TJ, Chua C, Falduto J (2001). Sex differences and opposite effects of stress on dendritic spine density in the male versus female hippocampus. *Journal of Neuroscience*.

[28] Paralikar V, Agashe M, Sarmukaddam S, Deshpande S, Goyal V, Weiss MG (2011). Cultural epidemiology of neurasthenia spectrum disorders in four general hospital outpatient clinics of urban Pune, India. *Transcultural Psychiatry*.

[29] Furukawa TA, Azuma H, Takeuchi H, Kitamura T, Takahashi K (2011). 10-year course of social adjustment in major depression. *International Journal of Social Psychiatry*.

[30] Rice NM, Grealy MA, Javaid A, Millan Serrano R (2011). Understanding the social interaction difficulties of women with unipolar depression. *Qualitative Health Research*.

[31] Fava M (2000). Introduction: optimizing treatment outcome in depression. *The Journal of Clinical Psychiatry*.

[32] Guidi J, Fava GA, Fava M, Papakostas GI (2011). Efficacy of the sequential integration of psychotherapy and pharmacotherapy in major depressive disorder: a preliminary meta-analysis. *Psychological Medicine*.

[33] Gottlieb L, Waitzkin H, Miranda J (2011). Depressive symptoms and their social contexts: a qualitative systematic literature review of contextual interventions. *International Journal of Social Psychiatry*.

[34] Leff J, Vearnals S, Brewin CR (2000). The London depression intervention trial. Randomised controlled trial of antidepressants v. couple therapy in the treatment and maintenance of people with depression living with a partner: clinical outcome and costs. *British Journal of Psychiatry*.

